# An Access Control Scheme Based on Blockchain and Ciphertext Policy-Attribute Based Encryption

**DOI:** 10.3390/s23198038

**Published:** 2023-09-23

**Authors:** Ronglei Hu, Ziwei Ma, Li Li, Peiliang Zuo, Xiuying Li, Jiaxin Wei, Sihui Liu

**Affiliations:** Department of Electronics and Communication Engineering, Beijing Electronic Science and Technology Institute, Beijing 100070, China; huronglei@sina.com (R.H.);

**Keywords:** ciphertext policy–attribute-based encryption (CP-ABE), privacy preserving, access control, blockchain, policy updating

## Abstract

Ciphertext policy–attribute-based encryption (CP-ABE), which provides fine-grained access control and ensures data confidentiality, is widely used in data sharing. However, traditional CP-ABE schemes often choose to outsource data to untrusted third-party cloud service providers for storage or to verify users’ access rights through third parties, which increases the risk of privacy leakage and also suffers from the problem of opaque permission verification. This paper proposes an access control scheme based on blockchain and CP-ABE, which is based on multiple authorization centers and supports policy updating. In addition, blockchain technology’s distributed, decentralized, and tamper-proof features are utilized to solve the trust crisis problem in the data-sharing process. Security analysis and performance evaluation show that the proposed scheme improves the computational efficiency by 18%, 26%, and 68% compared to previous references. The proposed scheme also satisfies the indistinguishability under chosen-plaintext attack (IND-CPA).

## 1. Introduction

With the advent of the big data era, data have become an important economic asset and a new factor of production permeating all walks of life. With increasing data resource sharing, incidents such as data theft and information leakage have occurred repeatedly. In May 2022, General Motors released a statement saying that some online customers had suspicious logins and that hackers had accessed some of their personal information through an online mobile application, including names, email addresses, mailing addresses, interest collections, search history, etc. In December of the same year, Azalea Motors suffered a server misconfiguration that led to the leakage of millions of user information and was subjected to a ransom demand of USD 2.25 million in Bitcoin equivalent. To prevent incidents like these, data owners often want to keep their data under control after they have been shared. Access control is an effective means to protect data security and control the flow and sharing of data. It explicitly allows or restricts the subject’s access to the object in a certain way, allowing users to access data within their legitimate permissions. If not, their operations are prohibited. Traditional access control cannot cope with the characteristics of a high degree of sharing and fast data circulation in the big data environment, and problems such as a single point of failure, difficulty in meeting the principle of minimum authorization, difficulty in dynamically adapting to changes in the environment, and difficulty in verifying policy authority have brought “shackles” to the development of access control technology.

The emergence of Attribute-Based Encryption (ABE) [1] brings a new solution to the above problem, which was first proposed by Sahai et al. in 2005, and subsequently developed into Key Policy–Attribute-Based Encryption (KP-ABE) [2] and Ciphertext Policy–Attribute-Based Encryption (CP-ABE) [3]. Data owners can customize access policies in CP-ABE and embed them in ciphertext. This approach not only realizes fine-grained and flexible access control but also solves the problems of traditional access control, which is challenging to satisfy the principle of minimum authorization and dynamically adapt to environmental changes. However, traditional CP-ABE schemes still have some drawbacks in practical applications. For example, in most CP-ABE schemes, with the help of a fully trusted attribute authorization authority, a single authorization authority needs to manage all users’ attributes and be responsible for the key generation and distribution process, which is a considerable workload. Moreover, if the single authorization center in these schemes fails or is maliciously attacked, many user attribute data will be exposed, and the whole system will be affected [4].

Blockchain technology [5], a widely emerging distributed-ledger-based technology, has become a reasonable choice for conducting trusted access control due to its decentralization, data tampering, traceability, non-falsification, and programmability. However, blockchain’s open and transparent nature makes the security and regulation of data stored in blockchain face a series of challenges [6,7]. If blockchain technology is combined with CP-ABE, the cryptographic mechanism of CP-ABE can ensure the confidentiality, privacy, and security of the data stored on the blockchain. In contrast, blockchain technology can provide functions like trusted authority verification and effective auditing for the existing CP-ABE-based schemes. For example, the programs in reference [8] achieve privacy protection and support the accountability of historical protocols through CP-ABE and blockchain technology. Reference [9] considers the advantages of CP-ABE and blockchain combined with cloud storage systems to share medical information effectively. Still, cloud servers storing a large amount of data can lead to privacy leakage and access control that policy permissions cannot credibly verify.

The leading platforms for blockchain applications are Bitcoin, Ethereum, and Hyperledger Fabric. Bitcoin is the blockchain technology’s prototype but lacks privacy protection and smart contracts. As a result, it cannot be used in complex situations. Ethereum is the public blockchain. However, any transaction on Ethereum requires payment. The public blockchain also has no restrictions on participants, who usually participate anonymously, making the public chain quite difficult to regulate. Fabric, as a representative of the consortium blockchain, requires participants to have explicit identifiers so that a malicious participant can be found directly. Therefore, Fabric is more secure than Ethereum. In addition, Fabric provides strong flexibility, pluggability, and scalability by modularizing the technologies of rights management, authentication, consensus mechanism, etc. Fabric can be more easily applied to complex scenarios by designing and developing smart contracts to execute various business logic.

Fabric can also design and develop smart contracts to implement different business logic, making it easier to apply to complex environments.

In summary, the existing schemes still have problems in access control, such as data leakage caused by semi-trusted cloud service providers, a non-transparent and untrustworthy verification of access privileges, an over-concentration of risk in single authorization centers, and overloaded computation. To solve the above problems, this paper proposes an access control scheme based on blockchain and CP-ABE, based on the characteristics of blockchain being de-trusted, decentralized, non-tamperable, and non-forgeable. The main contributions of this paper are as follows:

1. A new blockchain-based attribute-based cryptographic access control model is proposed. The introduction of blockchain in this model realizes the auditability of key parameter transmission and the trusted verification of data integrity, decryption correctness, etc., and solves the opaque verification problem of access rights caused by untrusted third parties. The combination of attribute-based encryption and blockchain guarantees that the data owner autonomously and effectively controls the circulation of shared data and protects the security of shared data.
2. An improved CP-ABE algorithm supporting access policy updates is proposed. This algorithm updates the access policy without disclosing the original encrypted data and realizes finer-grained and more flexible one-to-many access control with high efficiency and low overhead.
3. Using the threshold secret sharing algorithm to split the private key parameters of data visitors, the user private key needs to be calculated by multiple authorization centers collaboratively. Therefore, a single authorization center does not have the ability to generate user private keys. This method not only solves the problems of poor security, heavy computational burden, and unguaranteed data integrity of a single authorization organization, but also improves user privacy and key security.

## 2. Related Works

Currently, some research focuses on using a single authorization center for access control of attribute-based encryption in cloud computing environments. For instance, Li et al. [10] suggested a safe data-sharing system based on attribute encryption for users with restricted resources. By boosting the system’s public parameters and transferring a portion of the encrypted calculations to an offline state, this approach removed the majority of the calculation jobs. However, this scheme’s access strategy was limited to a description of positive and negative characteristics, leaving out the overall access control framework. Since we must completely rely on the single authorization center, if it is attacked, all the attributes will be accessed, thus lowering the security of the data stored in the cloud.

A solution with numerous authorization centers was created on the foundation of the conventional centralized single authorization agency solution in order to address the issues of low efficiency and inadequate security of a single authorization center. By expanding attribute sources and decentralizing the central master key, the attribute-based encryption scheme of the authorization center increased the variety of access tactics and encryption security. Sharma et al. [11] resolved the key escrow problems by using two authorities in the key generation process. Data owners and attribute authorities manage the key-related and user access policy details in a distributed manner. A new CP-ABE technique for multi-authorization agencies was also created by Gao et al. [12], which enhances user privacy and key security while significantly reducing the possibility of a single point of failure. A method called T-DPU-MCP-ABE (Traceable and Dynamic Policy Updating Multiauthority Attribute-based Encryption) was proposed by Ling et al. [13] in 2021. Data owners may frequently need to adjust the ciphertext access policy to meet diverse requirements. Updates to rules give data owners flexibility and enable them to fine-tune their encrypted data access restrictions for more precise control. A multi-authorization attribute-based encryption system with policy updating and concealing was suggested by Zhang et al. [14]. The untrusted issue with single attribute authorization is likewise resolved by multiple attribute authorities. Data owners can also easily and affordably adjust the access control policy. However, the above scenario relies on a fully trusted agent or a third-party central authority in addition to a semi-trusted multi-authorization center. In practical scenarios, fully trusted agents and third-party central organizations do not exist, and semi-trusted third parties bring new privacy issues. The multi-authorization center scheme proposed in this paper does not rely on a third party and can solve the trust problem in practical applications.

Blockchain technology [5] is an evolving technology with the advantage of decentralization, which is based on a distributed ledger. The issue of data in cloud storage that can be tampered with and whose integrity cannot be guaranteed has been resolved with the creation of blockchain technology [15]. Additionally, this technology is ideally suited to address issues related to ABE. Therefore, to better realize the secure and dependable large-scale exchange of vast data, researchers have recently integrated blockchain and attribute-based encryption technology. An independently revised key strategy ABE system was put forth by Guo et al. [16]. In addition, utilizing blockchain and distributed database technology protected the integrity of private healthcare data stored in the public cloud, preventing nefarious users or authorizers from tampering with private data from the internal cloud, thereby reducing misdiagnosis caused by tampered electronic health records. This scheme was managed by multiple attribute authorization centers, which was closer to the actual situation. To accomplish fine-grained access control to data, Zuo et al. [17] integrated blockchain with attribute-based encryption technology, making all user operations on the chain immutable and permanently maintained. In order to enable cloud security sharing without the usage of a trusted third party, blockchain was employed to authenticate user identities and manage their access privileges. This system prevented unauthorized users from accessing the data until user identification verification was complete. To protect data and attribute privacy, Gao et al. [18] introduced the ABE approach to the blockchain. Due to the strategy’s limited applicability to the compound order group, efficiency was low. A decentralized architecture was proposed by Vasishta et al. [19] to validate access requests through numerous authorities in a decentralized way using Hyperledger Fabric and Attribute-Based Access Control (ABAC). Reference [20] suggested putting ABAC into practice for decentralized authorization across numerous entities. Each organization in this implementation has a separate smart contract to add properties. A blockchain-based cloud data access authorization updating mechanism that supports keyword retrieval was proposed by Lei et al. [21]. According to reference [22], an industrial client can also validate the legitimacy of an IoT device’s distributor and manufacturer and grant permission for the IoT device to join their IoT network by issuing a network certificate. In reference [23], a multi-layer strategy uses blockchain technology for IoT device authentication and authorization. Some of the above research solutions use blockchain to solve only the cloud storage problem, and some only use blockchain for authorization, which does not involve whether the data owner can control the users who access the data. The proposed scheme in this paper uses the consortium blockchain fabric to verify the data in a trustworthy way and realizes transparent pre-verification of access rights by deploying smart contracts on the blockchain so that the data owner has the right to decide who can access the data and realizes true data security.

## 3. Access-Control-Scheme-Based Blockchain and CP-ABE

### 3.1. Main Parameters and Definitions

The main parameters involved in the scheme of this paper are shown in Table 1.

### 3.2. System Model

**Figure 1:**
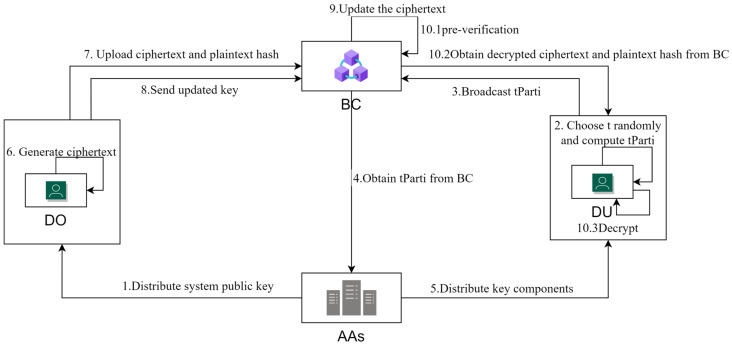
System model.

The system model proposed in this paper is shown in Figure 1, which includes four types of entities: Data Owner (DO), Data Visitor (DU), Attribute Authorization Center (${AA}_{S}$), and Blockchain (BC).

1. ${AA}_{s}$ computes the system public key and system master key and distributes the system public key to DO. 2. DU randomly selects the t-value and computes ${tPart}_{i}$. 3. DU broadcasts the computed ${tPart}_{i}$ to BC nodes. 4. When the data visitor DU needs to obtain its private key, the attribute authorization center ${AA}_{S}$ obtains ${tPart}_{i}$ from BC. 5. ${AA}_{S}$ distributes the private key component of DU obtained by substituting ${tPart}_{i}$ to DU. 6. DO generates the ciphertext by specifying a specific access policy. 7. DO then uploads the ciphertext and the hash value of the plaintext to BC. 8. If the access policy needs to be updated, DO sends the updated key to BC. 9. BC updates the ciphertext based on the updated key. 10.1 After DU obtains the key component from ${AA}_{S}$, BC pre-verifies whether DU has the access right to the data uploaded by DO. 10.2 If the verification passes, DU can obtain the ciphertext and the hash value of the plaintext from BC. 10.3 DU decrypts the ciphertext with its private key. In the above process, the entities undertake the following tasks (see Figure 1):

- ${AA}_{S}$: Organizations that manage attributes in the system. It manages the respective attributes and generates the master key MSK and system public key PK in step 1. In step 4, it obtains the slice of *t*, ${tPart}_{i}$, from the computation nodes in the blockchain and distributes the private key component of DU to the DU in step 5.
- DU: Users who want to access the data uploaded by the DO. In steps two and three, DU is responsible for computing ${tPart}_{i\in (1,n)}$ and broadcasting it to the blockchain. DU obtains the pre-verification result from the blockchain in part 10.1 of step 10. And if the DU has access to the data, the DU obtains the ciphertext and the hash values of the plaintext in part 10.2 of step 10, decrypts them, and verifies the correctness of $t^{\prime }$ using the private key of the DU in part 10.3. If required, DU can verify the correctness of decryption by querying the blockchain transaction.
- DO: The user who owns the data and specifies the attributes the visitor requires. The data owner encrypts the plaintext data using a symmetric key, specifies the access policy A for the data, encrypts the symmetric key to generate the ciphertext CT in step 6, and uploads the ciphertext and the data digest to the blockchain through a transaction in step 7. Based on the running results of the policy comparison algorithm deployed on the smart contract, DO selects the corresponding update key to send to the blockchain via the transaction in step 8.
- BC: Consortium blockchain fabric, a platform that ensures that data are stored and shared in a trusted manner. Users can publish and query transactions on the blockchain, providing storage services to secure data (${tPart}_{i}$ and ciphertext) and services for trusted authentication. In step 9, the blockchain performs the ciphertext update. The blockchain also does the 10.1 pre-verification part of the decryption phase.

### 3.3. Security Model

Choice Plaintext Attack (CPA) means that the attacker can select a plaintext message in addition to knowing the encryption algorithm to obtain the encrypted ciphertext, i.e., the attacker knows the chosen plaintext and the encrypted ciphertext. Still, they cannot directly break the key. Proof of security is performed by choosing a game of indistinguishability under a plaintext attack, and a game between an adversary and a challenger describes this game.

- Initialization phase. Challenger C runs the system initialization algorithm, inputs the security parameter λ, outputs the system public key PK and sends it to adversary A, and saves the system master key MSK. Adversary A selects the old access policy (A,ρ) and the new access policy $\left(A^{\prime },\rho^{\prime }\right)$ that it wishes to attack.
- Querying private key phase 1. Adversary A adaptively submits a series of attribute sets $U=(u_{1},u_{2},u_{3},\ldots \ldots u_{l})$ that do not satisfy the old and new access policies to challenger C. C runs the key generation algorithm to generate the corresponding key SK to sends it to adversary A.
- Challenge phase. Adversary A submits two equal-length messages $m_{0}$ and $m_{1}$ to challenger C. C randomly selects bϵ(0,1) and runs a cryptographic algorithm to encrypt $m_{b}$ under the old access policy P; challenger C performs a dynamic update of the access policy and generates an updated ciphertext based on the determination result of the old and the new access policies; C sends the updated ciphertext to adversary A.
- Querying private key phase 2. Similar to Phase 1, adversary A continues to submit attribute $u_{l}$ to challenger C for querying other attribute keys, with the stipulation that $u_{l}$ still does not satisfy the access policy P.
- Guessing phase. Adversary A gives a guess $b^{\prime }$ for b. The adversary wins the game if $b^{\prime }=b$, since any inactive adversary A can win this game with a probability $\frac{1}{2}$ by making a random guess on b. A’s advantage in this game is defined as
  $${Ad}_{vA}=|Pr\left[b^{\prime }=b\right]-\frac{1}{2}|$$

The goal of the adversary is to guess the value of b with a probability greater than $\frac{1}{2}$. If the above ${Ad}_{vA}$ can be ignored, this cryptosystem is considered indistinguishable under a chosen-plaintext attack and is called IND-CPA secure.

### 3.4. Operation Flow

**Figure 2:**
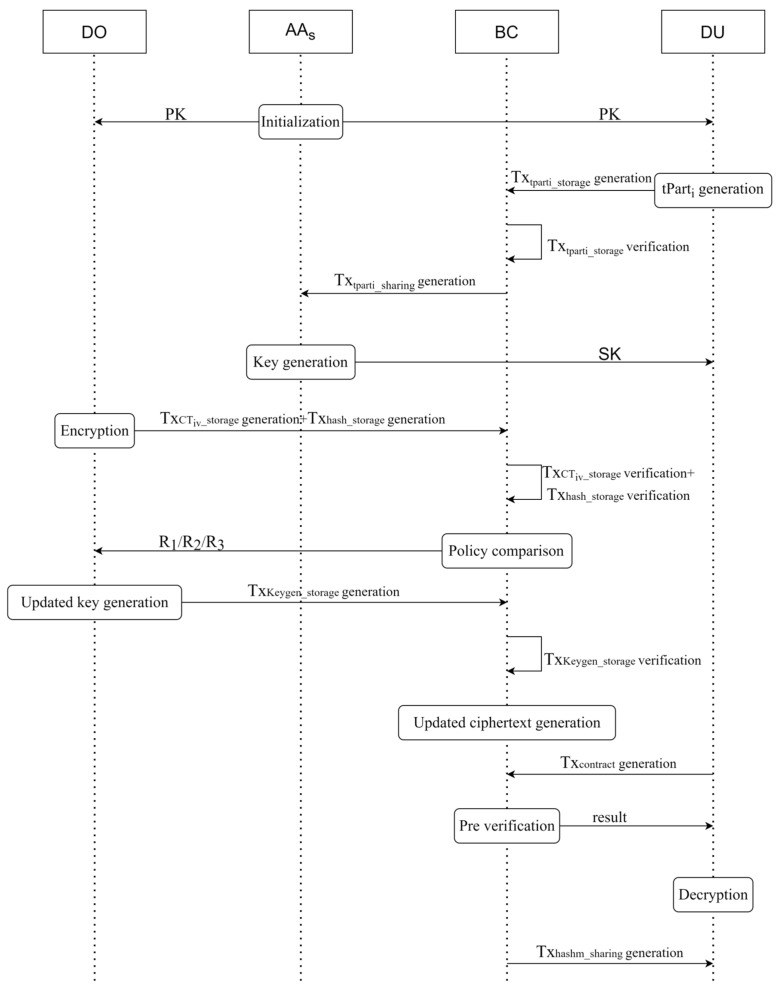
Scheme operation flow.

The operation flow of the data access control scheme based on blockchain and attribute-based encryption is shown in Figure 2, and the specific implementation details of this scheme are as follows.

### 3.5. Scheme Construction

#### 3.5.1. Initialization Stage

AAs executes the algorithm. The algorithm inputs the security parameter λ and outputs the system public key $PK=(G,G_{1},g,H,e\left(g,g\right),\alpha r,g^{\alpha },\alpha \beta )$ and the system master key MSK=(r,α,β) with randomly chosen $r,\alpha ,\beta \in Z_{p}$. The bilinear mapping $e:G\times G\to G_{1}$, where G and $G_{1}$ are cyclic groups of primes order p, and g is the generator of G. The hash function H maps an attribute in the system to an element in G. S denotes the set of attributes managed by the AAs.

#### 3.5.2. ${tPart}_{i}$ Generation Stage

The algorithm is executed by DU. The DU inputs random numbers (m,n) and $t\in Z_{p}$, and uses the threshold secret sharing algorithm to divide t into n slices of t*,* ${tPart}_{i\in (1,n)}$.

(1) $$f\left(x\right)=t+a_{1}x+\cdots +a_{m}x_{m}$$

(2) $${tPart}_{i}=f\left(x_{i}\right)$$

DU broadcasts the slices of t to the blockchain via the ${tPart}_{i}$ storage transaction, and nodes in the blockchain verify the transaction.

#### 3.5.3. ${Tx}_{{tPart}_{i}\_storage}$ Generation Stage

When Fabric processes each transaction, each link needs to verify the authority of the transaction information, and the application client invokes the certificate CA service through the SDK for registration and enrollment and obtains the identity certificate. So, DU, DO, and AAs also have their public–private key pairs in Fabric.

After generating the ${tPart}_{i}$ storage transaction shown in Algorithm 1, DU broadcasts the transaction to other nodes in the blockchain to verify it.

**Algorithm 1** ${Tx}_{{tPart}_{i}\_storage}$ generation transaction.**Input:** transaction id, ${tPart}_{i}$, public key ${PK}_{AAs}$ of AAs in the blockchain, the address of nodes ${adress}_{ComNodes}$, private key ${SK}_{DU}$ of DU in the blockchain **Output:**
${Tx}_{{tPart}_{i}\_storage}$ transactionfor 1≤i≤n  do    /*AAs encrypt _${tPart}_{i}$ with their public keys ${PK}_{AAs}$ in the blockchain*/    $E\left({tPart}_{i}\right)={Enc}_{{PK}_{AAs}}({tPart}_{i})$    /*DU compute the hash value of the transaction id, $E\left({tPart}_{i}\right)$ and the address of the nodes ${adress}_{ComNodes}$.*/    $s_{{tPart}_{i}}=hash\left\{id,E\left({tPart}_{i}\right),{adress}_{ComNodes}\right\}$    /*DU sign $s_{{tPart}_{i}}$ with their private keys ${SK}_{DU}$ in the blockchain*/    $sign={sign}_{{SK}_{DU}}(s_{{tPart}_{i}})$    ${Tx}_{{tPart}_{i}\_storage}=\left\{id,E\left({tPart}_{i}\right),{adress}_{ComNodes},sign\right\}$  return ${Tx}_{{tPart}_{i}\_storage}$

#### 3.5.4. ${Tx}_{{tPart}_{i}\_storage}$ Verification Stage

Each node receives the ${Tx}_{{tPart}_{i}\_storage}$ transaction; obtains the transaction id, $E\left({tPart}_{i}\right)$, and ${adress}_{ComNodes}$ from it; and compares it with the DU signed digest $s_{{tPart}_{i}}$ by computing the hash value $s_{{tPart}_{i}}^{\prime }$ of the three, as shown in Algorithm 2. If it returns true, it means the verification passes, and nodes in the blockchain will obtain $E\left({tPart}_{i}\right)$ from the ${Tx}_{{tPart}_{i}\_storage}$ transaction to keep it on behalf of the AAs.

**Algorithm 2**  ${Tx}_{{tPart}_{i}\_storage}$ verification transaction.**Input:**${Tx}_{{tPart}_{i}\_storage}$, public key ${PK}_{DU}$ of DU in the blockchain **Output:** verification resultsfor 1≤i≤n  do    /*Nodes compute the hash value of the transaction id, $E\left({tPart}_{i}\right)$ and the address of the nodes ${adress}_{ComNodes}$.*/    $s_{{tPart}_{i}}^{\prime }=hash\left\{id,E\left({tPart}_{i}\right),{adress}_{ComNodes}\right\}$    /*Countersign*/    $s_{{tPart}_{i}}={Compute}_{{PK}_{DU}}\left(sign\right)$    if $s_{{tPart}_{i}}^{\prime }=s_{{tPart}_{i}}$      return true    else      return false


#### 3.5.5. ${Tx}_{{tPart}_{i}\_sharing}$ generation Stage

In Algorithm 3, AAs obtains $E\left({tPart}_{i}\right)$ through the ${Tx}_{{tPart}_{i}\_sharing}=\left\{id,E\left({tPart}_{i}\right),{adress}_{AAs},sign\right\}$ transaction and decrypts $E\left({tPart}_{i}\right)$ with its own private key ${SK}_{AAs}$ to obtain ${tPart}_{i}$. The above three transactions coordinate with transferring ${tPart}_{i}$ from DU to AAs and, at the same time, record the transmission process of the actual key parameter ${tPart}_{i}$ on the blockchain. It reduces the security risks faced by ${tPart}_{i}$ in the process and realizes the traceability of key parameters.

**Algorithm 3**  ${Tx}_{{tPart}_{i}\_sharing}$ generation transaction.**Input:** transaction id, $E\left({tPart}_{i}\right)$, the address of the AAs ${adress}_{AAs}$, private key ${SK}_{ComNodes}$ of the nodes in the blockchain **Output:**
${Tx}_{{tPart}_{i}\_sharing}$ transactionfor 1≤i≤n  do    /*Nodes in the blockchain compute hash value*/    ${sh}_{{tPart}_{i}}=hash\left\{id,E\left({tPart}_{i}\right),{adress}_{AAs}\right\}$    /*Nodes sign ${sh}_{{tPart}_{i}}$ with their private keys ${SK}_{ComNodes}$ in the blockchain*/    $sign={sign}_{{SK}_{ComNodes}}({sh}_{{tPart}_{i}})$    ${Tx}_{{tPart}_{i}\_sharing}=\left\{id,E\left({tPart}_{i}\right),{adress}_{AAs},sign\right\}$  return ${Tx}_{{tPart}_{i}\_sharing}$


#### 3.5.6. Key Generation Stage

The algorithm is executed by the AAs that keep the ${tPart}_{i}$. The algorithm takes the set of user attributes $U=(u_{1},u_{2},u_{3},\ldots \ldots u_{l})$, ${tPart}_{i}$, PK, and MSK as inputs, and outputs the user’s private key SK. The AAs firstly reconstructs the value t secretly, and for each $u_{i}\in U$, the respective attribute authorization center computes the private key component $D_{i,1}$.

(3) $$g\left(x\right)=\prod_{i=1}^{m}\frac{x-x_{j}}{x_{i}-x_{j}}$$

(4) $$t=\sum_{i=1}^{m}{tPart}_{i}\cdot g\left(x_{i}\right)$$

(5) $$\forall u_{i}\in U,SK=\left\{D_{i,1},D_{2},D_{3},D_{4}\right\}=\left\{D_{i,1}={H\left(u_{i}\right)}^{rt},D_{2}=g^{\frac{1}{\beta }},D_{3}=g^{\alpha t},D_{4}=g^{r}\right\}$$

The user combines the above four key components to obtain $SK=\left\{D_{i,1},D_{2},D_{3},D_{4}\right\}$. The above private key generation stage has at least threshold value m attribute authorization centers for collaborative computation to secretly reconstruct the value t. Therefore, a single ${AA}_{i}$ does not have the ability to generate DU key components. When a single authorization center suffers from the attack, the security of the whole system will not be affected, which ensures that the DU private key will not be leaked.

#### 3.5.7. Encryption Stage

The encryption phase is performed by DO. This paper adopts the linear secret sharing schemes (LSSS) access structure. The algorithm takes PK, plaintext M, and k×l access matrix A as inputs, where ρ maps each row of the matrix to an attribute. The output is the ciphertext CT associated with the access structure A. The algorithm chooses the random vector $\vec{v}=(s,v_{2},\ldots v_{l})\in Z_{p}^{l}$, $v_{2},\ldots v_{l}$ for sharing the secret s. $\lambda_{x}=A_{x}{\vec{v}}^{T}$ is calculated. The random vector $\vec{\omega }=(0,\omega_{2},\ldots \omega_{l})\in Z_{p}^{l}$ is chosen and $\omega_{x}=A_{x}{\vec{\omega }}^{T}$ is chosen, where $A_{x}$ represents the vector corresponding to the *x*th row of matrix A. $L_{x}=\left\{x|\rho (x)\in S\right\}$ is defined, $z_{x}\in Z_{p}$ is chosen at random, and the following is computed:

(6) $$C_{M}=M{e\left(g,g\right)}^{sr}$$

(7) $$\begin{array}{cc} \forall x\in \left[1,k\right],CT & =\left({CT}_{iv},{CT}_{v}\right)\\ & =\left\{{{(C}_{x,1},C_{x,2})}_{x\in \left[1,k\right]},{{(C}_{x,3}{,C}_{x,4},C_{M})}_{x\in \left[1,k\right]}\right\}\\ & =\left\{\begin{array}{c} C_{x,1}={H\left[\rho \left(x\right)\right]}^{{rz}_{x}},C_{x,2}=g^{\alpha z_{x}}g^{\alpha },C_{x,3}={H\left[\rho \left(x\right)\right]}^{\alpha \beta }g^{\omega_{x}},\\ C_{x,4}=g^{\lambda_{x}},C_{M}=M{e\left(g,g\right)}^{sr} \end{array}\right\} \end{array}$$

In this paper, the shared data are subjected to a blockchain-based decentralized trusted verification of access rights and encryption/decryption correctness, and the ciphertext CT components are classified into two categories: pre-verification ciphertext ${CT}_{iv}$ and decryption ciphertext ${CT}_{v}$. The ciphertext CT is uploaded to the blockchain and verified through the ${Tx}_{{CT}_{iv}\_storage}$ generation transaction. In the encryption phase, DO also needs to calculate the hash value hashm of the plaintext M, upload it to the blockchain through the ${Tx}_{hashm\_storage}$ transaction, and verify it. The ${Tx}_{{CT}_{v}\_storage}$ generation transaction and ${Tx}_{hashm\_storage}$ generation transaction are similar to the ${Tx}_{{tPart}_{i}\_storage}$ generation transaction and ${Tx}_{{CT}_{v}\_storage}$ verification transaction, respectively; and the ${Tx}_{hashm\_storage}$ verification transaction is similar to the ${Tx}_{{tPart}_{i}\_storage}$ verification transaction.

#### 3.5.8. Policy Comparison Stage

In Algorithm 4, if the DO needs to update the access policy, the DO invokes the policy comparison algorithm deployed in the smart contract, and this algorithm outputs the results $R_{1}$, $R_{2}$, and $R_{3}$ by determining the type of the new policy attribute $\rho^{\prime }\left(j\right)$.

**Algorithm 4** Policy comparison algorithm.**Input:** old policy (A,ρ), new policy $\left(A^{\prime },\rho^{\prime }\right)$ $\mathrm{Output}:\;R_{x\in (1,2,3)}$
for $j\in [1,k^{\prime }]$ do  if $\rho^{\prime }(j)$ exist in A then    if $L_{x}\neq \emptyset$ and $\exists x\in L_{x}$,$\rho (x)=\rho^{\prime }\left(j\right)$      add (j,x) into $R_{1}$      delete x from $L_{x}$
    else      ∀x∈[1,k],$\rho (x)=\rho^{\prime }\left(j\right)$      add (j,x) into $R_{2}$  else    add (j,0) into $R_{3}$


#### 3.5.9. Updated Key Generation Stage

At the updated key generation stage, DO inputs PK, selects the updated key ${UK}_{x\in (A,B,C)}$ according to the output of the policy comparison algorithm, and constructs new random vectors ${\vec{v}}^{\prime }\in Z_{p}^{l^{\prime }}$ and ${\vec{\omega }}^{\prime }\in Z_{p}^{l^{\prime }}$, where the first terms are s and 0, respectively. DO computes $\lambda_{j}=A_{j}^{\prime }{{\vec{v}}^{\prime }}^{T}$ and $\omega_{j}=A_{j}^{\prime }{{\vec{\omega }}^{\prime }}^{T}$. The generated policy updated key ${UK}_{m}$ is sent to the blockchain and verified by the ${Tx}_{UpKeygen\_storage}$ transaction, which is similar to the ${Tx}_{{tPart}_{i}\_storage}$ transaction.

$$\forall j\in \left[1,k^{\prime }\right],\left(j,x\right)\in R_{1}:{UK}_{1}=\left\{{UK}_{1,A}=g^{\omega_{j}-\omega_{x}},{UK}_{1,B}=g^{\lambda_{j}-\lambda_{x}}\right\}\;\forall j\in \left[1,k^{\prime }\right],\left(j,x\right)\in R_{2}:{UK}_{2}=\left\{a_{j},{UK}_{2,A}=g^{{\omega_{j}-a_{j}\omega }_{x}},{UK}_{2,B}=g^{{\lambda_{j}-a_{j}\lambda }_{x}}\right\},$$

where $r_{j}={r_{x}a}_{j}$,$\alpha_{j}=\alpha_{x}a_{j}$ and

$$\forall j\in [1,k^{\prime }],(j,x)\in R_{3}:{UK}_{3}=\left\{\begin{array}{c} {UK}_{3,A}={H\left[\rho^{\prime }\left(j\right)\right]}^{r_{j}z_{j}},{UK}_{3,B}=g^{\alpha_{j}z_{j}}g^{\alpha_{j}},\\ {UK}_{3,C}={H\left[\rho^{\prime }\left(j\right)\right]}^{\alpha_{j}\beta_{j}}g^{\omega_{j}},{UK}_{3,D}=g^{\lambda_{j}} \end{array}\right\}$$

#### 3.5.10. Updated Ciphertext Generation Stage

After receiving the policy updated key ${UK}_{x\epsilon (1,2,3)}$, the blockchain inputs the PK and the old ciphertext CT and updates the ciphertext as follows.

- If ${UK}_{1}$,

$$C_{j,1}^{\prime }=C_{x,1}={H\left[\rho^{\prime }\left(j\right)\right]}^{r_{j}z_{j}}\;C_{j,2}^{\prime }=C_{x,2}=g^{\alpha_{j}z_{j}}g^{\alpha_{j}}\;C_{j,3}^{\prime }=C_{x,3}\cdot {UK}_{1,A}={{H\left[\rho (x)\right]}^{\alpha_{x}\beta_{x}}g}^{\omega_{x}}g^{\omega_{j}-\omega_{x}}={H\left[\rho^{\prime }\left(j\right)\right]}^{\alpha_{j}\beta_{j}}g^{\omega_{j}}\;C_{j,4}^{\prime }=C_{x,4}=g^{\lambda_{x}}\cdot {UK}_{1,B}={g^{\lambda_{x}}g}^{\lambda_{j}-\lambda_{x}}=g^{\lambda_{j}}$$

- If ${UK}_{2}$,

$$C_{j,1}^{\prime }={{(C}_{x,1})}^{a_{j}}={H\left[\rho \left(x\right)\right]}^{{a_{j}r}_{x}z_{x}}={H\left[\rho^{\prime }\left(j\right)\right]}^{r_{j}z_{j}}\;C_{j,2}^{\prime }={{(C}_{x,2})}^{a_{j}}=g^{{\alpha_{x}z}_{x}a_{j}}g^{\alpha_{x}a_{j}}=g^{{\alpha_{j}z}_{j}}g^{\alpha_{j}}\;C_{j,3}^{\prime }={{(C}_{x,3})}^{a_{j}}\cdot {UK}_{2,A}={H\left[\rho \left(x\right)\right]}^{{a_{j}\alpha }_{x}\beta_{x}}g^{{a_{j}\omega }_{x}}\cdot g^{{\omega_{j}-a_{j}\omega }_{x}}={H\left[\rho^{\prime }\left(j\right)\right]}^{\alpha_{j}\beta_{j}}\;C_{j,4}^{\prime }={{(C}_{x,4})}^{a_{j}}{UK}_{2,B}=g^{a_{j}\lambda_{x}}g^{{\lambda_{j}-a_{j}\lambda }_{x}}=g^{\lambda_{j}},$$

where $r_{j}={a_{j}r}_{x}$ and $\alpha_{j}=\alpha_{x}a_{j}$.

- If ${UK}_{3}$,

$$C_{j,1}^{\prime }={UK}_{3,A}={H\left[\rho^{\prime }\left(j\right)\right]}^{r_{j}z_{j}}\;C_{j,2}^{\prime }={UK}_{3,B}=g^{\alpha_{j}z_{j}}g^{\alpha_{j}}\;C_{j,3}^{\prime }={UK}_{3,C}={H\left[\rho^{\prime }\left(j\right)\right]}^{\alpha_{j}\beta_{j}}g^{\omega_{j}}\;C_{j,4}^{\prime }={UK}_{3,D}=g^{\lambda_{j}}$$

The blockchain is authorized to re-encrypt the ciphertext, and the policy updated key only reveals the relationship between the old and new access policies. It does not disclose any information about the encrypted data.

#### 3.5.11. Decryption Stage

The decryption phase consists of two parts: pre-verification and decryption. Pre-verification verifies whether the DU has access rights to the data, and the DU invokes the smart contract through the contract generation transaction ${Tx}_{contract}$ and obtains the partial ciphertext before decryption to verify the access rights. If the DU attribute sets satisfy the access policy, the algorithm will output the correct result. Otherwise, it will output ⊥, as shown in Algorithm 5.

**Algorithm 5** Pre-verification algorithm.$\mathrm{Input}:\;{CT}_{iv}$, $D_{i,1}$, $D_{3}$, PK Output: result or ⊥
for 1≤i≤n  do    $e\left(C_{x,2},D_{i,1}\right)=e\left(g^{\alpha z_{x}}g^{\alpha },{H\left(u_{i}\right)}^{rt^{\prime }}\right)={e(g,H\left(u_{i}\right))}^{\alpha z_{x}{rt}^{\prime }}{\cdot e(g,H\left(u_{i}\right))}^{\alpha rt^{\prime }}$    $e\left(g^{\alpha },D_{i,1}\right)=e\left(g^{\alpha },{H\left(u_{i}\right)}^{rt^{\prime }}\right)={e(g,H\left(u_{i}\right))}^{\alpha rt^{\prime }}$    $e\left(C_{x,1},D_{3}\right)=e\left({H\left[\rho (x)\right]}^{{rz}_{x}},g^{\alpha t^{\prime }}\right)={e(H(\rho (x),g)}^{rz_{x}\alpha t^{\prime }}$    if $\frac{e\left(C_{x,2},D_{i,1}\right)}{e\left(C_{x,1},D_{3}\right)\cdot e\left(C_{x,1},D_{3}\right)}=1$      $result=\left\{e\left(C_{x,2},D_{i,1}\right),e\left(C_{x,1},D_{3}\right)\right\}$      return result    else      return ⊥

After verifying the access rights, the fully trusted DU receives the result, obtains the decrypted ciphertext ${CT}_{v}$ from the blockchain through the ${Tx}_{{CT}_{v}\_sharing}$ transaction, and decrypts it with the private key. In the decryption phase, DU inputs PK, DU’s private key SK, and value t. If the value of t is the same as the value of $t^{\prime }$ after collaborative computation by m attribute authorization centers in Keygen phase, the following computation is performed:

(8) $$e\left(C_{x,4},D_{4}\right)=e\left(g^{\lambda_{x}},g^{r}\right)={e\left(g,g\right)}^{\lambda_{x}r}$$

(9) $${e\left(C_{x,3},D_{i,2}\right)}^{rt}={e\left({H\left[\rho \left(x\right)\right]}^{\alpha \beta }g^{\omega_{x}},g^{\frac{1}{\beta }}\right)}^{rt}={e(H(\rho (x),g)}^{\alpha rt}\cdot {e\left(g,g\right)}^{\frac{{rt\omega }_{x}}{\beta }}$$

(10) $$\tilde{C}=\frac{e\left(C_{x,2},D_{i,1}\right)\cdot {e\left(g,g\right)}^{\lambda_{x}r}}{{e(H(\rho (x),g)}^{\alpha rt}\cdot {e\left(g,g\right)}^{\frac{{rt\omega }_{x}}{\beta }}\cdot e\left(C_{x,1},D_{3}\right)}$$

In the above process, $t^{\prime }=t$ proves that m semi-trusted attribute authorizations correctly compute the value $t^{\prime }$, and the legitimate DU obtains the correct key component. If $\tilde{C}=\frac{{e(g,g)}^{\lambda_{x}r}}{{e(g,g)}^{\frac{{rt\omega }_{x}}{\beta }}}$), then DU chooses the constant $c_{x}\in Z_{p}$ that satisfies $\sum c_{x}A_{x}=(1,0,\ldots ,0)$ and computes

(11) $$\frac{C_{M}}{\prod {\tilde{C}}^{c_{x}}}=M^{\prime }$$

This process generates plaintext $M^{\prime }$. The user DU encrypts the plaintext $M^{\prime }$ obtained from the decryption algorithm with the help of the SHA256 algorithm and compares its hash value $hashm^{\prime }$ with the hashm obtained through the blockchain ${Tx}_{hashm\_sharing}$ transaction. If $hashm^{\prime }=hashm$, then the decryption is correct and the data are complete.

## 4. Proof of Security

**Definition** **1.** *(The Decisional q-Parallel Bilinear Diffie–Hellman Exponent problem): Choose a group* G *of prime order p according to the security parameter. Let* $a,s,b_{1},\ldots ,b_{q}\epsilon Z_{q}$ *be chosen at random and* g *be a generator of* G*. If an adversary is given,* y=

$$g,g^{s},g^{a},\ldots ,g^{\left(a^{q}\right)},g^{\left(a^{q+2}\right)},\ldots ,g^{\left(a^{2q}\right)}\;\forall_{1\leq j\leq q}g^{s\cdot b_{j}},\;g^{\frac{a}{b_{j}}},\ldots ,g^{\left(\frac{a^{q}}{b_{j}}\right)},g^{\left(\frac{a^{q+2}}{b_{j}}\right)},\ldots ,g^{\left(\frac{a^{2q}}{b_{j}}\right)}\;\forall_{1\leq j,k\leq q,k\neq j}g^{\frac{asb_{k}}{b_{j}}},\ldots ,g^{\frac{a^{q}sb_{k}}{b_{j}}}$$

It must remain difficult to distinguish ${e(g,g)}^{a^{q+1}s}\epsilon G_{T}$ from a random element in $G_{T}$. An algorithm B that outputs zϵ(0,1) has an advantage ε in solving the decisional q-parallel BDHE in G if

$$\left|Pr\left[B\left(y,T={e(g,g)}^{a^{q+1}s}\right)=0\right]-Pr\left[B\left(y,T=R\right)=0\right]\right|\geq \varepsilon$$

We say that the (decision) q parallel-BDHE assumption holds if no polytime algorithm has a non-negligible advantage in solving the decisional q-parallel BDHE problem.

**Theorem** **1.** *Suppose an adversary* A *can attack this scheme with a non-negligible advantage ε; in probabilistic polynomial time. Then, a challenger* C *can solve the q-Parallel BDHE hypothesis with an advantage of* ε/2.

**Proof of** **Theorem** **1.** The challenger picks two multiplicative cyclic groups G and $G_{T}$, the generator g, and the bilinear mapping $e:G\times G\to G_{T}$. Set a q-Parallel BDHE challenge with randomly chosen $b\epsilon \left\{0,1\right\}$, $R\epsilon G_{T}$, and take $Z={e(g,g)}^{a^{q+1}s}$ if b=0; otherwise, take T=R.

- Initialization phase.

The challenger C obtains the access structure $\left(A^{*},\rho^{*}\right)$ that the adversary A wishes to challenge, and $A^{*}$ is a $k^{*}\times l^{*}$ matrix. C chooses the random number $\alpha^{\prime }\in Z_{p}$ such that $e\left(g^{\alpha },g^{a^{q}}\right){e(g,g)}^{\alpha^{\prime }}={e(g,g)}^{\alpha }$, with the implication that $\alpha =\alpha^{\prime }+a^{q+1}$.

For each x in $A_{x}$, C chooses $m_{x}$ at random. Let X be the set of x satisfying $\rho^{*}\left(x\right)=x$. It can be sought that

$$H\left(A_{x}\right)=g^{m_{x}}\prod_{x\in X}g^{\frac{aA_{x,1}^{*}}{b_{i}}}\cdot g^{\frac{a^{2}A_{x,1}^{*}}{b_{i}}}\cdot \ldots \cdot g^{\frac{a^{l^{*}}A_{x,l}^{*}}{b_{i}}}$$

The above expression $g^{m_{x}}$ is randomly distributed, so $H\left(A_{x}\right)$ is also randomly distributed. If X=∅, $H\left(A_{x}\right)=g^{m_{x}}$.

- Querying private key phase 1.

Adversary A adaptively submits a set of attributes U not satisfying $\left(A^{*},\rho^{*}\right)$ to ask for the private key. Challenger C chooses the random number $r\in Z_{p}$ and finds the vector $\vec{\omega }=(\omega_{1},\omega_{2},\ldots \omega_{l})\in Z_{p}^{l^{*}}$ with the first term $\omega_{1}=1$, $\vec{\omega }\cdot A_{x}^{*}=0$ for $\rho^{*}(x)\in U$. Define $K^{\prime }=g^{r}\prod_{x=1}^{l^{*}}{\left(g^{a^{q+1-x}}\right)}^{\omega_{x}}=g^{t}$, where $t=r+\omega_{1}a^{q}+\omega_{2}a^{q-1}+\ldots +\omega_{l}a^{q-l^{*}+1}$.

Challenger C cannot model the unknown term $g^{\frac{a^{q+1}}{b_{i}}}$, so it must be ensured that the $K_{x}$ expression does not contain a term of the form $g^{\frac{a^{q+1}}{b_{i}}}$. Challenger C computes $K=g^{\alpha^{\prime }}g^{ar}\prod_{x=2}^{l^{*}}{\left(g^{a^{q+2-x}}\right)}^{\omega_{x}}$.

If x∈U and there is no x such that $\rho^{*}\left(i\right)=x$, one can make $K_{x}=K^{\prime m_{x}}$.

If x∈U, there is more than one x such that $\rho^{*}\left(i\right)=x$, and all equations of the form $g^{\frac{a^{q+1}}{b_{i}}}$ can be cancelled out by $\vec{\omega }\cdot A_{x}^{*}=0$. Let X be the set of x satisfying $\rho^{*}\left(x\right)=x$. C constructs $K_{x}$ according to the following equation:

$$K_{x}=K^{\prime m_{x}}\prod_{i\in X}\prod_{j=1}^{l^{*}}{\left\{{{(g}^{\frac{a^{j}}{b_{i}}})}^{r}\prod_{\begin{array}{c} u=1,\ldots l^{*}\\ u\neq j \end{array}}{\left(g^{\frac{a^{q+1+j-n}}{b_{i}}}\right)}^{\omega_{u}}\right\}}^{A_{i,j}^{*}}$$

- Challenge phase.

The adversary A submits two equal-length challenge messages $m_{0}$ and $m_{1}$ to the challenger C. C chooses at random b∈(1,0) and the random numbers $y_{2}^{\prime },\ldots ,y_{l^{*}}^{\prime }$ and uses $\vec{v}$ for secret sharing on s.

$$\vec{v}=(s,sa+y_{2}^{\prime },sa^{2}+y_{3}^{\prime },\ldots ,sa^{l-1}+y_{l^{*}}^{\prime })\in Z_{p}^{l^{*}}$$

In addition, C randomly selects $z_{1}^{\prime },\ldots ,z_{l}^{\prime }$ to generate the challenge ciphertext ${CT}^{*}$ as follows:

$$C_{M}=m_{b}\cdot Z\cdot e\left(g^{s},g^{\alpha^{\prime }}\right)\;C_{x,1}=H^{rz_{l}^{\prime }}\left[\rho (x)\right],\;C_{x,2}=g^{\alpha z_{l}^{\prime }}g^{\alpha },\;C_{x,3}=g^{\alpha \beta }g^{\omega_{x}^{\prime }},\;C_{x,4}=g^{\lambda_{x}^{\prime }}$$

- Querying private key phase 2.

Similar to Phase 1.

- Guessing phase.

The adversary A outputs a guess $b^{\prime }$ for b. If $b^{\prime }=b$, challenger C outputs $\mu^{\prime }=0$, denoting $Z={e(g,g)}^{a^{q+1}s}$; otherwise, challenger C outputs $\mu^{\prime }=1$, denoting Z=R. A’s advantage in that game is defined as ε.

If μ=0, A obtains a valid ciphertext and $Pr\left[b^{\prime }=b|\mu =0\right]=\frac{1}{2}+\varepsilon$;

C guesses that $b^{\prime }=b$ when $\mu^{\prime }=0$ and $Pr\left[\mu^{\prime }=\mu |\mu =0\right]=\frac{1}{2}+\varepsilon$;

If μ=1, A obtains invalid information and $Pr\left[b^{\prime }\neq b|\mu =1\right]=\frac{1}{2}$;

C guesses that $b^{\prime }\neq b$ when $\mu^{\prime }=1$ and $Pr\left[\mu^{\prime }=\mu |\mu =1\right]=\frac{1}{2}$.

Thus, the advantage of C in solving the deterministic hypothesis q-Parallel BDHE problem is

$$\begin{array}{cc} {Ad}_{vA} & =Pr\left[b^{\prime }=b\right]-\frac{1}{2}\\ & =\frac{1}{2}Pr\left[\mu^{\prime }=\mu |\mu =0\right]+\frac{1}{2}Pr\left[\mu^{\prime }=\mu |\mu =1\right]-\frac{1}{2}\\ & =\frac{1}{2}\left(\frac{1}{2}+\varepsilon \right)+\frac{1}{2}\times \frac{1}{2}-\frac{1}{2}\\ & =\frac{\varepsilon }{2}\;\;\;\;□ \end{array}$$

Based on the q-Parallel BDHE hypothesis, the challenger has a non-negligible advantage, proving that the scheme proposed in this paper is IND-CPA-safe under the q-Parallel BDHE hypothesis.

## 5. Performance Analysis

### 5.1. Functional Comparison

This section presents a comparative analysis of the schemes of reference [3,14,24] and this paper in terms of six aspects: access structure, multiple authorization centers, policy update, pre-validation, correctness verification, and blockchain, as shown in Table 2. The symbols × and √ represent whether the scheme has the function. Table 2 shows that reference [14] implements decryption correctness verification, and this paper and reference [24] implement decentralized trusted verification based on blockchain, which implements a pre-verification of access rights and verification of decryption correctness, respectively. Among these three schemes, reference [24] has only one authorization center to manage all users’ attributes and key distribution, which is a heavy workload and faces trust crises. Zhang et al. [14] and the scheme proposed in this paper also implement the access policy update function under the premise of multiple authorization centers. However, the access structure of reference [14] adopts a simple AND gate, which supports only the AND form when expressing the access policy and cannot express access policies with more complex logical structures, and is also limited in realizing fine-grained access control. Meanwhile, the multi-authorization center in the system designed in reference [14] needs a fully trusted third-party central authority to distribute global parameters for it, but in some application scenarios, the fully trusted third party does not exist, in which case the semi-trusted multi-authorization center may distribute incorrect private keys to legitimate data visitors, and the practical application scenarios of reference [14] are greatly limited. This is the reason why this paper designs the data visitor to provide the t-value in advance, which ensures that the semi-trusted multi-authorization center still generates the correct private key for the legitimate user in the absence of a third-party fully trusted central authority. In addition, the blockchain introduced in this paper’s scheme also guarantees that the data delivered under the de-trusted condition are not tampered with. In summary, the scheme in this paper realizes the above essential features simultaneously, solves the trust problem of access control systems in practical application scenarios, and is functionally superior to other schemes.

### 5.2. Comparison of Computation

Table 3 demonstrates the computational overhead of [3,14,24] and this paper, where $n_{u}$ and $n_{p}$ represent the number of attributes and the number of policies, respectively; ${exp}_{G}$ and ${exp}_{G_{1}}$ represent the exponential operation in G and $G_{1}$, respectively; and e is the bilinear operation. Table 3 shows that this paper’s scheme has the lowest total computational overhead in key generation, encryption, and decryption.

### 5.3. Efficiency Analysis

This experiment is based on Ubuntu 22.04.2 LTS and the Charm-crypto library, running on a computer (Intel(R) Core(TM) i5-7300HQ CPU @ 2.50 GHz,8.00 GB RAM). In this paper, we use a 160-bit group of elliptic curves in hyper-singular curve $y=x^{3}+x$ over a 512-bit finite field. The experimental data are taken as the average value of the data obtained from 20 runs. This section compares this scheme with the existing schemes mainly in key generation time, encryption time, and decryption time.

**Figure 3:**
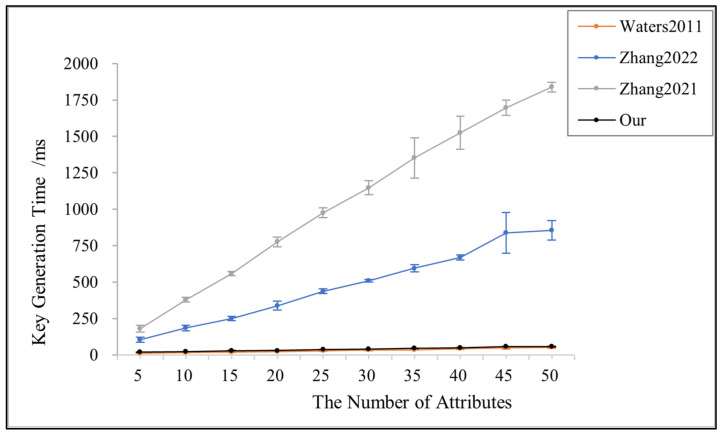
Key generation time for references [3,14,24] and this paper.

Figure 3 shows the variation in key generation time with the number of attributes in [3,14,24] and the proposed scheme in this paper. Since reference [24] needs to create the vector y for decryption, it has the highest computational overhead and the longest key generation time. The key generation time of this paper’s scheme is very low and basically does not vary with the number of attributes, which is close to that of reference [3]. However, the key generation process in reference [3] is managed by only one authorization center, which is inefficient and less secure.

**Figure 4:**
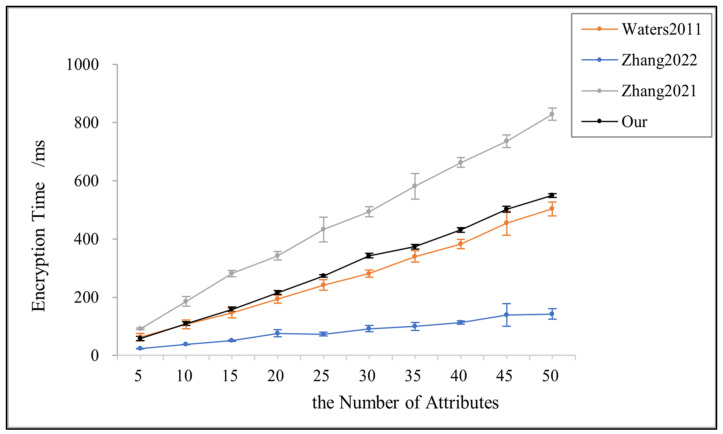
Encryption time for references [3,14,24] and this paper.

Figure 4 illustrates the variation in encryption time with the number of attributes in [3,14,24] and the proposed scheme in this paper. All the results are linearly related to the number of attributes. Reference [14] is based on the AND gate access structure; its scheme sacrifices more fine-grained access policies for a lower cost encryption time. Reference [3] does not support the policy update function that facilitates the data owner to perform access control. However, in the actual access process, to dynamically adapt to changes in the environment and keep the shared data in a controllable range, the data owner must be able to formulate and update the access policy according to the demand dynamically and flexibly. This paper uses the linear secret sharing scheme, which is practical in that it can be implemented in any monotonic access structure despite the slightly higher encryption cost and can be used for policy updates without compromising user privacy.

**Figure 5:**
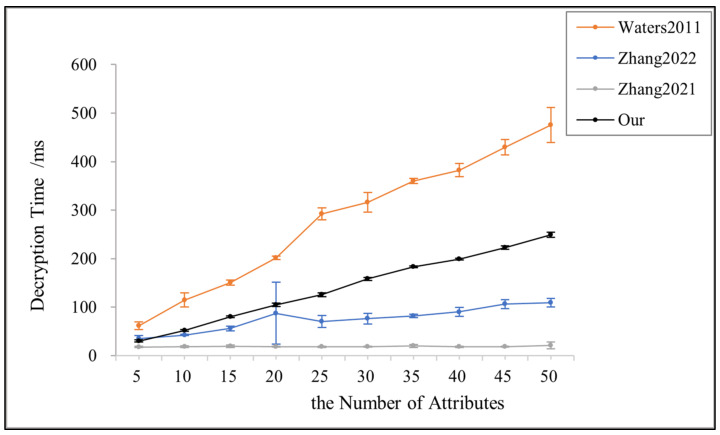
Decryption time for references [3,14,24] and this paper.

Compared with the existing schemes, this paper, in order to ensure that legitimate users will not obtain the wrong key components provided by the semi-trusted attribute authorization centers, chooses to verify the correctness of the $t^{\prime }$ computed by the m attribute authorization centers by the value t provided by the DU in the decryption phase, increasing the ${exp}_{G}$ operation and making the decryption stage slightly more computationally intensive than in reference [14] and reference [24], as shown in Figure 5. However, in reference [14], only linear operations are required for decryption because this scheme supports only AND gate structures. Reference [24], on the other hand, outsources most of the decryption computation to the blockchain, which significantly increases the cost of using the blockchain.

**Figure 6:**
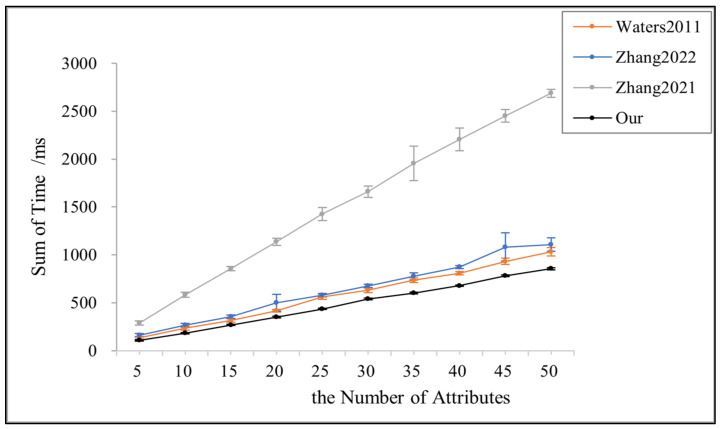
Time of key generation + encryption + decryption for references [3,14,24] and this papers.

In order to comprehensively and holistically compare the performance of the four schemes, this paper finds the sum of the key generation time, the encryption, and the decryption time of each scheme, as shown in Figure 6. Figure 6 shows that this paper’s scheme spends the lowest sum of time in the key generation, encryption, and decryption stages, consistent with each scheme’s theoretical and computational overhead results demonstrated in Table 3.

### 5.4. Blockchain Network Simulation

In the blockchain simulation experiment, this paper builds the Fabric consortium blockchain based on Ubuntu 22.04.2 LTS, which uses the PBFT consensus algorithm. The chaincode used in the scheme is developed with the Golang language. In this paper, the Caliper tool measures the transaction throughput and latency of the generation and verification transactions under different numbers of transactions. The test object of this experiment is a randomly selected node from the blockchain network, and the test content is the generation transaction and the verification transaction with a concurrency of 100–1000.

**Figure 7:**
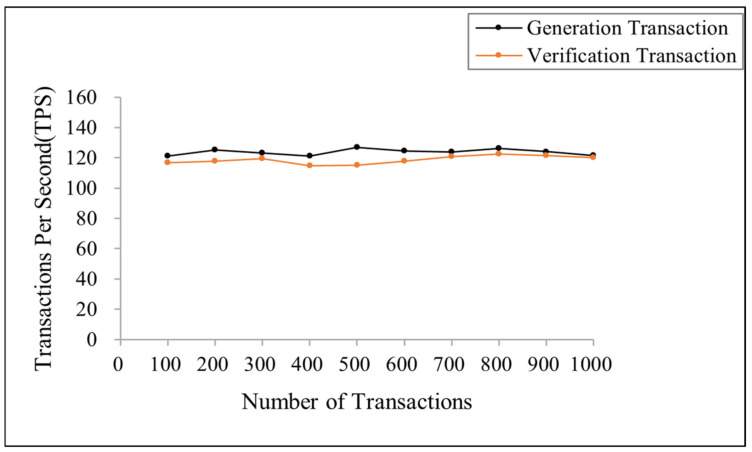
Throughput of the generation and verification transaction under different transaction numbers.

Throughput is the speed at which the blockchain ledger receives transactions, measured by the number of transactions executed per second. Figure 7 shows the throughput of two types of transactions for different transaction numbers in the blockchain. From Figure 7, as the number of transactions increases, the throughput of a single node remains near 120, indicating that the scheme proposed in this paper has good scalability.

**Figure 8:**
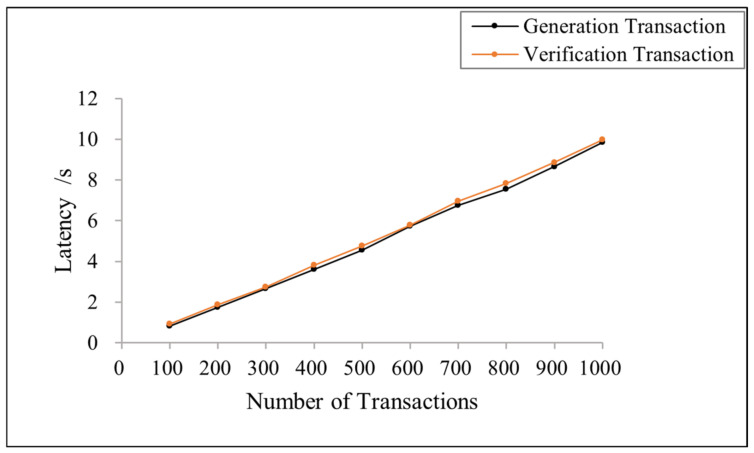
Latency of the generation and verification transaction under different transaction numbers.

Figure 8 shows the variation in transaction latency with the number of transactions in the proposed scheme. The results show that the latency of both types of transactions increases linearly with the number of transactions because increasing the number of transactions causes the waiting queue to become longer. Nonetheless, the generation transaction and verification transaction time remain within 10 s with a concurrency of 100–1000. Each node has a faster transaction speed, which provides a high-performance service.

## 6. Conclusions

This paper proposes a multi-authorization access control scheme based on blockchain and CP-ABE, adopting a matrix access structure and supporting policy update functions to realize flexible and fine-grained access control. In this paper, blockchain is combined with attribute-based encryption, where the data owner embeds the access policy into the ciphertext data through attribute-based encryption and later uploads it to the blockchain. The blockchain records the transmission process of the data visitor’s private key to pre-verify the data visitor’s access rights. The combination of the two not only ensures the confidentiality and security of the data on the blockchain, but also realizes the auditable and highly transparent access process, which solves the problems of traditional access control, such as single-point failure, difficulty in meeting the principle of minimum authorization, difficulty in dynamically adapting to changes in the environment, and difficulty in verifying the access rights of the policy.

## Figures and Tables

**Table 1:** Main parameters and definitions.

Parameters	Definitions
${AA}_{s}$	Multiple attribute authorization centers
${AA}_{i}$	Single attribute authorization center
DU	Data visitors
DO	Data owners
PK	system public key
MSK	System Master Key
SK	user private key
S	Attribute sets managed by the Attribute Authorization Center
e()	bilinear mapping
$G,G_{1}$	prime cyclic group
${tPart}_{i\in (1,n)}$	Users secretly share key shares
${Tx}_{X}$	Transactions about X in the blockchain
${adress}_{X}$	Address of X in the blockchain
${PK}_{X}$	Public key of X in the blockchain
${SK}_{X}$	Private key of X in the blockchain
${Enc}_{{PK}_{X}}(Y)$	Encrypt Y with the public key of X in the blockchain
$hash\left\{Y\right\}$	Hash operation on Y
${sign}_{{SK}_{X}}(Y)$	Sign Y with the private key of X in the blockchain
${Compute}_{{PK}_{X}}\left(Y\right)$	Countersign Y with the public key of X in the blockchain
M	Plaintext
CT	Ciphertext
${CT}_{iv}$	Pre-verification ciphertext
${CT}_{v}$	Decrypted ciphertext
$L_{x}$	Policy Index Set
hashm	plaintext hash
$A_{x}$	The vector corresponding to the xth row of matrix A
$\rho^{\prime }\left(j\right)$	Attribute in line j of the policy
$R_{x\in (1,2,3)}$	Policy Comparison Determination Results
${UK}_{x\epsilon (1,2,3)}$	Policy Updated Key

**Table 2:** Comparison of the performance of different schemes.

Scheme	Blockchain	Pre-Verification	Correctness Verification	Multi-Authorization Center	Policy Update	Access Structure
[3]	×	×	×	×	×	LSSS
[14]	×	×	√	√	√	AND
[24]	√	√	√	×	×	LSSS
Scheme of this paper	√	√	√	√	√	LSSS

**Table 3:** The computational overhead of different schemes.

Scheme	Keygen	Enc	Dec	Keygen+Enc+Dec
[3]	$\left(n_{u}+2\right){exp}_{G}$	$\left(3n_{p}+1\right){exp}_{G}+{exp}_{G_{1}}$	$n_{p}{exp}_{G_{1}}+(2n_{p}+1)$e	$\left(n_{u}+3n_{p}+3\right){exp}_{G}+\left(n_{p}+1\right){exp}_{G_{1}}+(2n_{p}+1)$e
[14]	$\left(3n_{u}+2\right){exp}_{G}$	$\left(n_{p}+3\right){exp}_{G}+n_{p}{exp}_{G_{1}}$	4e	$\left(3n_{u}+n_{p}+5\right){exp}_{G}+n_{p}{exp}_{G_{1}}+4e$
[24]	$5n_{u}{exp}_{G}$	$\left(5n_{p}+2\right){exp}_{G}+2{exp}_{G_{1}}$	${exp}_{G}+(n_{p}+1)e$	$\left(5n_{u}+5n_{p}+3\right){exp}_{G}+2{exp}_{G_{1}}+(n_{p}+1)e$
Scheme of this paper	$\left(n_{u}+3\right){exp}_{G}$	$5n_{p}{exp}_{G}+{exp}_{G_{1}}$	${exp}_{G}+2n_{p}e$	$\left(n_{u}+5n_{p}+4\right){exp}_{G}+{exp}_{G_{1}}+2n_{p}e$

## Data Availability

Data sharing is not applicable to this article.
